# APP-guided assessment of acetabular defects in hip revision arthroplasty: a structured approach to a complex situation

**DOI:** 10.1007/s00402-021-04270-8

**Published:** 2021-11-30

**Authors:** Max Jaenisch, Dieter Christian Wirtz, Hendrik Kohlhof, Martin Gathen, Koroush Kabir, Sebastian Koob, Tom Rainer Jansen

**Affiliations:** Department of Orthopedics and Trauma Surgery, University Hospital Bonn, Venusberg Campus 1, 53127 Bonn, Germany

**Keywords:** Acetabular, Bone defect, ADC, App, Application, Hip, Arthroplasty, Revision

## Abstract

**Introduction:**

Acetabular defect recognition and classification remains a challenging field of practice for orthopedic surgeons. Recently, the Acetabular Defect Classification (ADC) has been introduced to provide a reliable, reproducible and intuitive classification system. In order to improve ease of use and efficiency of the ADC, a browser-based application has been created. We hypothesized that the ADC application can improve rating performance of non-specialists (medical students) to achieve good inter- and intra-rater agreement and will compare favorable to the results of specialists (experienced surgeons) without the help of the application.

**Materials and methods:**

The ADC is based on the integrity of the acetabular rim and the supporting structures. It consists of four main types of defects ascending in severity. These defects are further subdivided in A–C, narrowing down defect location. 80 randomized radiographs were graded according to ADC by three non-specialists (medical students) with help of the ADC application and by three specialists (orthopedic surgeons) without help of the application to evaluate the difference in inter-rater agreement between groups. To account for intra-rater agreement, the rating process was repeated after a reasonable wash-out period.

**Results:**

Inter-rater and intra-rater agreement within the non-specialist group rated lower when compared to the specialist group while still falling into the good agreement range. The student group presented with *k* values of 0.61 for inter-rater agreement and 0.68 for intra-rater agreement, while the surgeon group displayed *k* values of 0.72 for inter-rater agreement and 0.83 for intra-rater agreement.

**Conclusion:**

The app-guided assessment of acetabular defects offers a promising innovative approach to simplify complex situations. It makes the challenging field of acetabular revision arthroplasty more approachable especially for less experienced surgeons and offers insight and guidance in the planning stage as well as intra-operative setting.

## Introduction

Modern total hip arthroplasty is considered to be the operation of the century, offering pain relief, advanced mobilization and an increased overall quality of life to a vast number of patients [1, 2]. While implantation rates are predicted to increase all over the developed world, due to the limited durability and survival of implants, revision cases tend to accumulate as well. Most cases of hip revision arthroplasty are accompanied by acetabular bone defects of varying degree. Depending on the severity of the damage to the remaining bone stock, primary stable implantation of the revision component and a favorable long-term outcome are demanding to achieve. Detailed and proper defect recognition and meticulous pre-operative planning are essential. To facilitate pre-operative grading of the expected bone loss, numerous acetabular defect classification systems have been established [3–6]. Recently, the Acetabular Defect Classification—short ADC—has been introduced to provide a reliable, reproducible and intuitive classification system, which offers a clear therapeutic guideline [7]. Even though the structured design facilitates intuitive use, acetabular defects present a complex and difficult field of practice and especially for unexperienced surgeons, ways to lower the introductory hurdle are more than welcome.

The omnipresent mobile device commonly known as “smart phone” has altered the way we interact with each other and how we consume information. Many studies have confirmed a significant increase in the number of smart phone users and the time being spent on mobile devices per day [8–10]. The introduction of customizable applications, which can be accessed through app stores or various internet browsers, offer the possibility of individualization and ubiquitous access to web-based information and innovative technologies [11]. Therefore, daily usage for various private and professional applications such as online banking or different modes of communication have become common place [12].

Medical applications are on the rise as well and their number and availability are constantly increasing. While an unified nomenclature is still lacking, most available applications are described as “health”, “lifestyle” or “care”-apps and are mostly aimed for individual adoption by private end users. Nevertheless, especially young surgeons in the field of Orthopedics and Trauma Surgery use smartphone apps daily in their clinical practice and the importance in the near future is projected to increase [13].

Due to the corona pandemic a steep increase in web-assisted patient care can be observed in clinical practice. Especially the utilization of telemedical examinations to reduce human contact and prevent a further spreading of the corona virus has increased significantly [14]. In these cases, medical applications such as virtual goniometers may be helpful to increase accuracy of a telemedical examination [15–17].

To further improve the ease of use and efficacy of the ADC, a browser-based application appears to be a natural evolution and has been developed to lead the user along the pre-operative radiographic grading process. To the authors’ knowledge, no system is available that applies an established acetabular defect classification system with the practical benefits of a browser-based application. App-guided evaluation of complex defect situations could offer a vast array of potential benefits, such as increased efficacy and a steeper learning curve. The ADC has been proven to offer a reliable pre-operative grading which corresponds closely with the encountered intra-operative findings [7]. Therefore, the following publication will exclusively focus on evaluating the improvement in grading experience generated through an web-based application.

We hypothesized that the ADC application can improve rating performance of non-specialists (medical students) to achieve good inter- and intra-rater agreement according to Landis and Koch, and will compare favorably to the results, which experienced orthopedic surgeons achieved without the help of the application [18].

## Materials and methods

### Study design

From a sample of 211 patients who underwent acetabular revision for various reasons with exchange of at least the acetabular component, a randomized sample of 80 pre-operative radiographs, after power analysis, was selected. All radiographs were screened for sufficient quality to allow for a reliable grading. To enable blinded assessment any identifying features were removed, and the images were anonymized by numerical coding. Radiographic assessment consisted of pelvis a.p. standing and involved hip axial. Radiographic analysis and grading were carried out using IMPAX EE (Agfa HealthCare GmbH, Bonn, Germany).

Raters were recruited and divided into two different groups. The first group (specialists, S) consisted of experienced orthopedic surgeons in the field of hip revision arthroplasty. The second groups consisted of medical students (non-specialists, nS). None of the raters had any prior knowledge of the ADC or were involved in the creation.

Each rater received a teaching session consisting of an overview of the classification system and the supervised evaluation of ten random cases, which did not include any of the tested 80 cases. A scoring sheet was distributed. Only the nS group received an introduction to the ADC application and was allowed to use it during the teaching session and further rating.

Radiographic evaluation was carried out at two different stages with a wash-out period of two weeks in between to avoid bias due to memorization. All images were relabeled and randomized prior to the second evaluation.

### Browser-based application and classification system

**Fig. 1:**
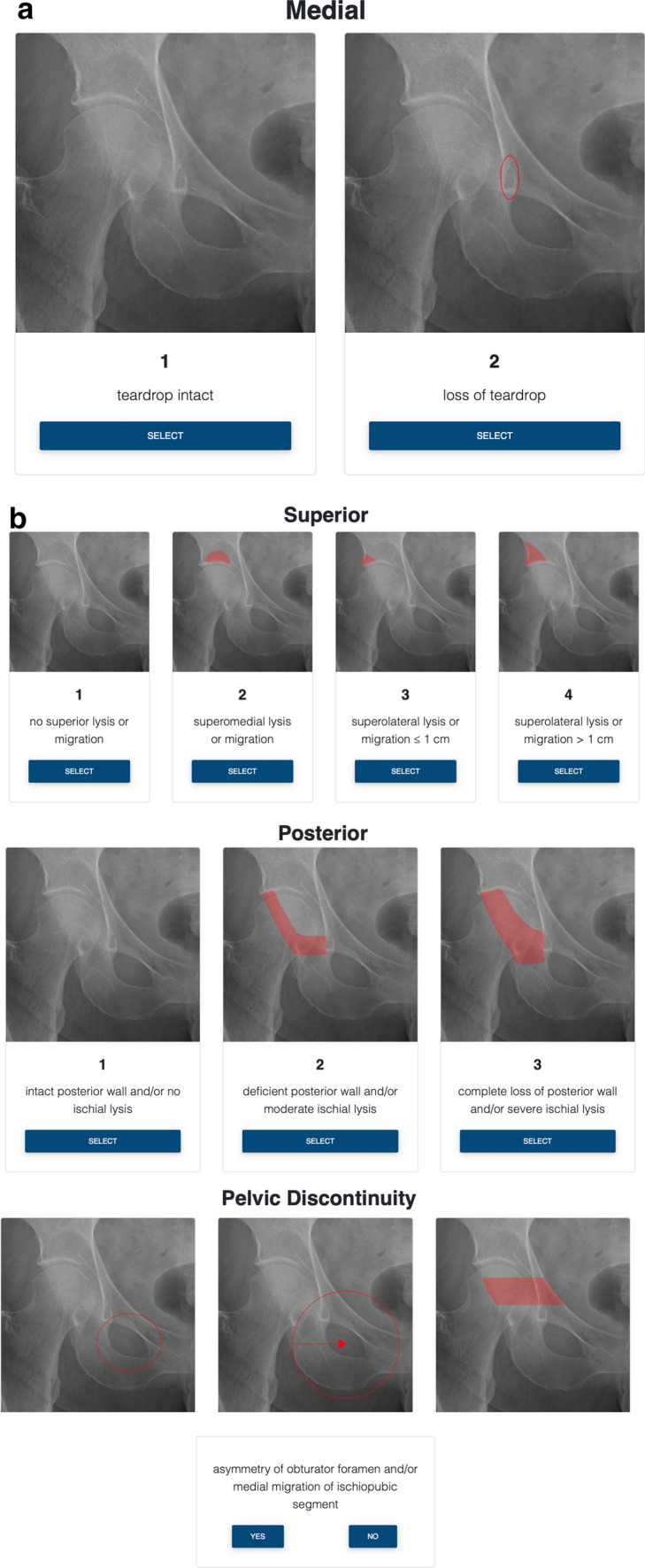
The ADC application is designed as a walk-through, guiding the end user through a radiographic evaluation of an acetabular defect. Due to the condensation of information by depicting a complex, three-dimensional bone defect on a two-dimensional native radiograph, structured and detailed evaluation is essential to achieve a reliable grading. In all grading steps a native a.p. radiograph of a right-sided, healthy hip joint is provided for comparison. **a** Beginning with the medial portion of the acetabulum, the end user can evaluate the integrity of the “teardrop”, a radiographic phenomenon resulting in the overlay of different bone surface encompassing the medial wall. The teardrop is marked with a red oval ellipse. For options, either “teardrop intact” or “loss of teardrop” can be selected. **b** In the next grading step, the superior aspect of the acetabular rim can be evaluated. The region of interest is clearly marked by a red color overlay. Options extend from “no superior lysis or migration”, over “superomedial lysis or migration”, and “superomedial lysis or migration < 1 cm”, to “superolateral lysis or migration > 1 cm. **c** On the following page the posterior aspect of the acetabulum is evaluated. The region of interest is clearly marked by a red color overlay. Possible choices are: “intact posterior wall and/or no ischial lysis”, “deficient posterior wall and/or moderate ischial lysis” and “complete loss of posterior wall and/or severe ischial lysis”. **d** At the last grading step direct or indirect signs of pelvic discontinuity need to be assessed. If any signs of pelvic discontinuity are present, such as “asymmetry of obturator foramen and/or medial migration of ischiopubic segment” (compare to the first and second marked image) or a visible fracture line can be observed (compare to the third marked image), the user can choose “yes” and continue

The browser-based application was built using Angular (Version 6, Google LLC, Mountain View, California, USA), a TypeScript-based open-source web application framework. The front-end design was built using Bootstrap (Version 4, Twitter Inc., San Francisco, California, USA). Cordova Apache (Version 4.5.5, Adobe Inc., San José, California, USA) was used to transform the web app to a native iOS application. All data are temporarily stored in the internal storage of the device and are deleted after each run time. No data is stored on the web server. The app itself is designed as a guided walk-through. Each sub-item of the acetabular defect characteristics can be chosen by a simple-selection button. Once a sub-item for each characteristic is chosen, the defect category can be calculated. After calculation, an example image of the chosen acetabular defect and a treatment option are displayed. Figure 1a–d displays the individual steps of the application work flow.

The Acetabular Defect Classification (ADC) is based on the integrity of the acetabular rim and the supporting structures. It consists of four main types of defects ascending in severity, with an additional subdivision into a, b and c. The following defect descriptions are extracted from the original publication introducing the ADC [7].

#### Type 1 defects

Type 1 defects are characterized as contained defects with the acetabular rim remaining intact and only showing cancellous bone defects in different locations according to subdivision. A 1A defect displays randomly distributed cancellous defects, which respect the superomedial aspect of the acetabulum, as well as the medial wall, leaving these structures intact. A 1B defect exhibits additionally to defects already described for a lysis of the superomedial acetabulum. A 1C defect displays a deficiency of the medial wall, which does not affect the anterior or posterior column.

#### Type 2 defects

Type 2 defects demonstrate a non-contained defect of the acetabular rim in addition to cancellous bone defects. The defect measures below or equal 10 mm and is considered as non-structural. For 2A the rim defect affects the superolateral portion while in 2B the posterior column is deficient. A type 2C defect is a combination of A and B and displays a defect including the full weight-bearing portion of the rim. Because of its measurement below 10 mm, it is also considered as non-structural.

#### Type 3 defects

**Fig. 2:**
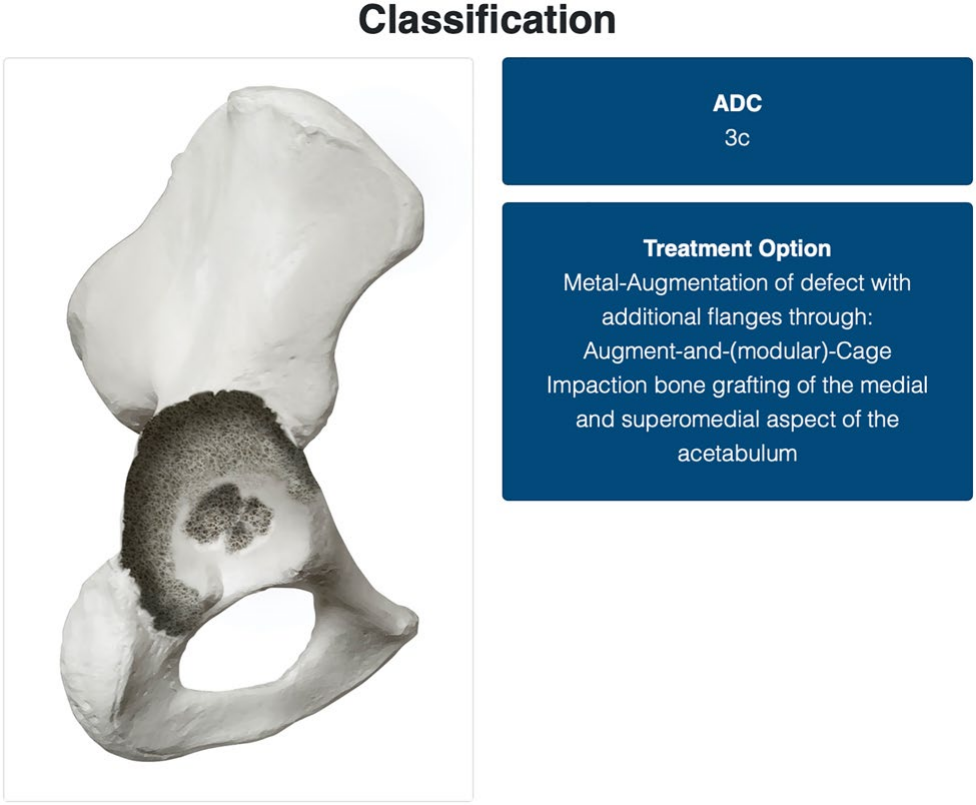
The depiction of one of the possible results. In this case, in the field “ADC”, a 3b defect has been classified. Possible implant choices are provided in the “Treatment Option” field. Each therapeutic recommendation is specifically tailored to the evaluated defect and based on a thorough review of the literature and expert opinion. On the left side of the figure, an anatomical representation of the defect is provided to further convey detailed understanding of the bone morphology

Type 3 defects possess non-contained, structural defects over 10 mm of the acetabular rim. The subdivision follows the same structure as for the type 2 defects with A, including the superior aspect, B, the posterior column and C being a combination of both. An illustration of a type 3 C defect is displayed in Fig. 2.

#### Type 4 defects

Type 4 defects involve pelvic discontinuity and are the most severe cases of acetabular bone loss. There is a disruption between the bone stock of the ischium and the ilium. The anterior and posterior columns are rendered non-supportive. The subclassification in A, B and C allow for evaluation of the therapeutic options by taking the superior bone support into consideration. For A, the superior bone stock is considered supportive, for B a non-structural superior rim defect under/equal 10 mm is described and for C, a structural superior rim defect over 10 mm accompanies the pelvis discontinuity.

### Statistical analysis

For the statistical analysis, IBM SPSS Statistics 1.0.0.1131 (IBM Inc., Armonk, New York, USA) was utilized. The level of significance was set at *p* < 0.05 and the confidence interval at 95%. To account for inter-rater reliability in the process of comparing ordered categorical data with more than two raters, Fleiss kappa was used. Through the utilization of Cohens kappa intra-rater reliability was calculated and compared through the mean kappa of all raters. Interpretation of kappa values was achieved through the agreement scale described by Landis and Koch. Kappa values exceeding 0.80 indicate excellent agreement, between 0.61 and 0.8 indicate good agreement, between 0.41 and 0.60 indicate moderate agreement, between 0.21 and 0.4 indicate fair agreement and between 0.20 and below indicates poor agreement [18].

## Results

**Fig. 3:**
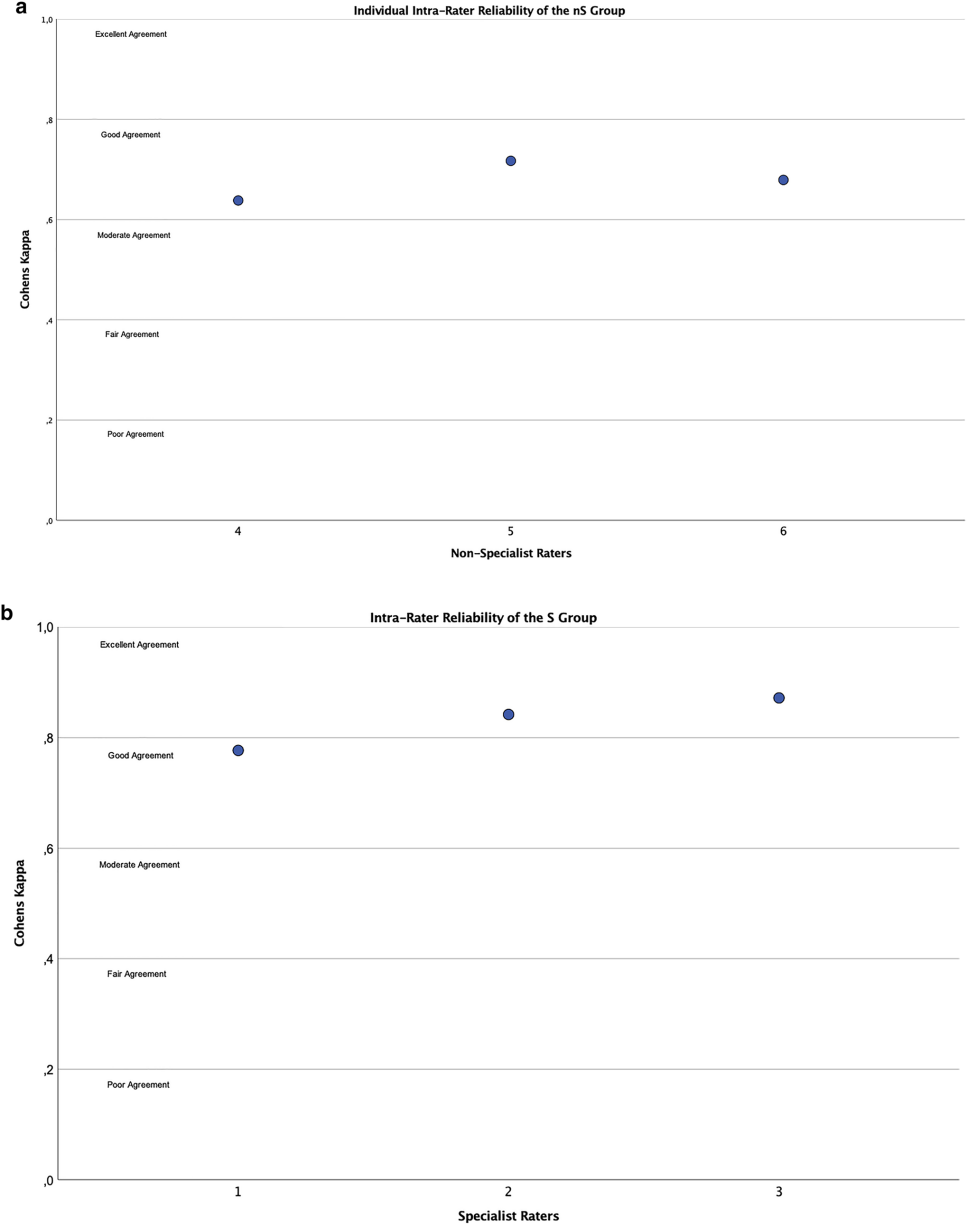
The display of the individual k values for intra-rater reliability of the S group (**a**) and the nS group (**b**). All values fall at least in the good agreement range with two raters in the S group achieving excellent agreement. The different agreement ranges are labeled and divided by horizontal lines to provide an interpretative context to the *k* values. Y-axis features Cohens kappa and X-axis the individual rater ID 1–5 of specialist and non-specialist raters

Inter-rater agreement of the nS group was evaluated at a *k* value of 0.611 ± 0.022, which differed significantly from the inter-rater agreement of the S group (*p* < 0.001), which was evaluated at a *k* value of 0.721** ± **0.023. Both results fall into the good agreement range as defined by the scale of Landis and Koch [18]. For the control of the reproducibility of the ADC applications impact on the quality of rating, intra-rater agreement has been examined after a wash-out period of two weeks. The nS group achieved a *k* value of 0.678 ± 0.040, while the surgeon group achieved a *k* value of 0.830 ± 0.049. There was once more a significant difference between both *k* values with the nS group registering lower than the S group (*p* < 0.001). Both values fall above the good agreement range with the nS group even displaying excellent agreement. The individual values for intra-rater reliability are illustrated in Fig. 3a, b.

**Fig. 4:**
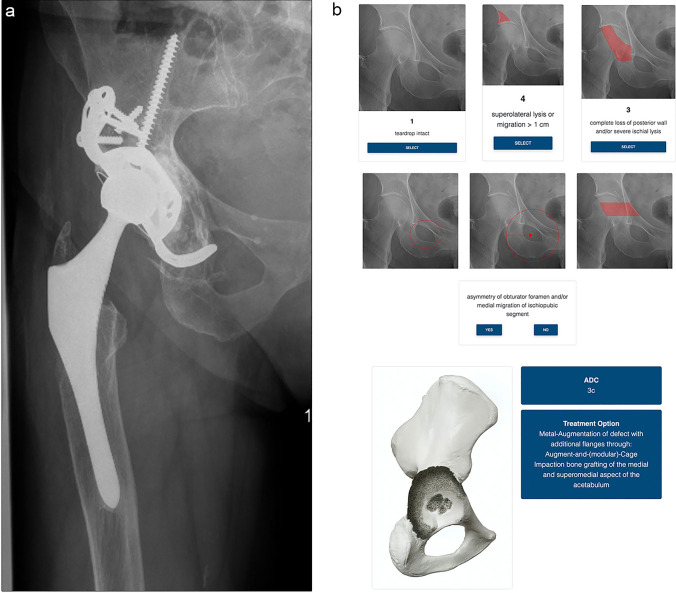
The presentation of the app-guided classification of a pre-operative X-ray of a 67-year-old, female patient with multiple previous operations of the affected hip. **a** shows the pre-operative AP pelvis view, loosening of the acetabular cage with superolateral migration and consecutive breakage of the screws on the cranial strap; **b** lists the selected subdivisions within the application: medial: the teardrop appears intact due to the superolateral migration; superior: the superolateral migration ensures a superior defect, removal of the fractured screws and bony debridement will further increase defect size, a superior defect of > 1 cm is to be expected; posterior: with the implant still in place radiopaque material limits the visualization of the posterior acetabular wall, cloudy osteolysis of the visible aspects indicate the presents of a bony defect, which should be selected accordingly. To further evaluate the posterior acetabular wall a CT scan can be helpful; pelvic discontinuity: the presented X-ray displays no indicators to suspect pelvic discontinuity. an ADC 3C defect with a treatment option is displayed

The feedback provided by the included raters (nS) conveyed a positive attitude and underlined the ease of use and an appreciation for a structured approach to a complex situation. Through the process of this evaluation, no technical issues such as failures to boot, slow servers and loading times or issues in coding were reported. The users displayed a steep learning curve and a quick adaption rate to the operating mode. During the conduct of this study, the web-based application could be utilized thorough out various operating system including Apple iOS, iPadOS and OSX (Apple, Cupertino, California, USA) and Windows 7, 8, 9 and 10 (Microsoft Corporation, Redmond, Washington, USA) and Android Open Handset Alliance (Google Limited Liability Company, Mountain View, California, USA) (Fig. 4).

## Discussion

The utilization of smartphone and mobile applications is projected to increase in the medical community [19–21]. While mostly younger and less experienced surgeons in the field of orthopedic and trauma surgery use their smartphone on a regular basis in clinical practice, surgeons with > 15 years of experience have shown to employ frequent use of their mobile devices as well [13, 20]. Though the majority of “health” applications are generated for non-medical end users, content provided for medical professionals is projected to increase [22, 23]. Considering the current trend of rapidly increasing smartphone use in the orthopedic community, the creation of mobile applications to aid the professional end users in their clinical practice appears to be a natural evolution [19].

Possible applications for mobile devices are nearly endless and include limited but instrumental tasks like goniometers, or simple but data-secure messaging tools and extend to complex classification systems and surgical reference guides as described below.

Acetabular bone loss in cases of revision hip arthroplasty has been a well-researched scientific field and many classification systems have been introduced as an attempt to manage the complexity [3–6]. In the literature, classification systems such as introduced by Paprosky et al., Gross et al. and the AAOS display varying reliability mostly ranging from moderate to poor [24]. While clinically and methodically well thought through, most classification systems are complex and appear overwhelming, especially when adopted by inexperienced surgeons. To provide a more reliable and easily adoptable classification system, the ADC has been introduced recently [7]. While presenting with good inter- and intra-rater agreement in the initial publication, it contains all clinically relevant defect morphologies while not being overly simplified. To further increase the ease of use, a conversion to a mobile application has been carried out resulting in this publication. Through our results, we were able to show that non-specialist raters (medical students) were able to achieve sufficient results in a complex setting, such as acetabular defects, when compared to experienced orthopedic surgeons. To the knowledge of the authors, no other acetabular defect classification system has been converted to a mobile application and therefore direct comparison is currently not possible.

While the conversion of complex classification systems into mobile applications has not yet been widely adopted in the field of orthopedic and trauma surgery, there is a remarkably similar example in the literature. The group around Riouallon created a smartphone application to aid the utilization of the complex Letournel classification system for acetabular fractures. Congruent to our own findings, the group discovered a high accuracy even for inexperienced raters through usage of the provided application. In addition, Riouallon observed a significant reduction in average interpretation time when the app was used, which has not been evaluated in this study but would be an interesting question for future research [22].

Another famous and widely adopted example is the browser-based application AOSR (Arbeitsgemeinschaft Osteosynthese Surgery Reference) created by the AO Foundation which helps with the classification of various fractures all over the human and even animal body and offers surgical guidance as well [25]. An assessment of the accuracy of the AO Spine Thoracolumbar Classification as part of the ASOR has been recently conducted by de Araujo Ono and colleagues. They succeeded in showing that residents improved their ability to recognize and classify thoracolumbar spine fractures through app-usage. Therefore, they concluded that their findings reinforce the importance of mobile applications in medical education and clinical practice [26]. The results of both publications support our findings.

The group around Dittrich recently conducted a large survey among orthopedic and trauma surgeons (*n* = 836) in order to investigate the current opinion regarding acceptance, future prospects and risks of the integration and use of smartphones in medical care [13]. Their results coincide strongly with our own aims in the creation of the ADC application. The group discovered that mostly younger and less experienced doctors were prone to regular smartphone use in their clinical practice. The ADC application, while free to use for anyone, is especially aimed at less experienced surgeons entering the field of acetabular reconstruction in order to ingrain a structured and reproducible guideline for diagnostic evaluation from the beginning. To achieve wide-spread adoption and regular usage, Dittrich found that an intuitive usability is considered favorable, which is produced by the structured algorithm of the ADC application and its walk-through-like design. Applications that are free of charge were also favored, which corresponds with the distribution mode of our application. Two of the major concerns mentioned in the publication by Dittrich were the perceived risk of data misuse and the danger of using untrustworthy apps. As for the first concern, the data storage of the ADC applications is limited to the utilized device and is eradicated once the session has ended. No data is stored on web-based servers. As for the fear of utilizing an untrustworthy application, it is worth noting, that the ADC in itself has been validated by the original publication of this group and therefore can be considered “safe to use” [7].

Our study presents the following limitations: the group of selected raters has not been randomized and was chosen by chance through employment at our institution for the S group and through university-mandated fellowships for the nS group. The number of selected raters is limited and therefore, results achieved by a larger cohort might differ. In this publication only the direct comparison between results of the two different groups has been evaluated. An actual improvement of the medical students (nS) through the app has not been examined, but it is safe to assume that an unaided approach of a non-specialist rater in the task of acetabular defect classification would yield lower k values for both inter- and intra-rater reliability. However, we strongly encourage future research by other groups in order to further evaluate our algorithm and application and gladly provide more detailed methods on request. Lastly, it is important to note that the web-based application merely offers a guided walk-through of the ADC. Visual and lyrical cues aim to increase ease of use of a complex classification system. Known deficiencies of acetabular defect recognition and classification, such as defect obscurrence by radio-opaque material, cannot be addressed by this type of application.

## Conclusion

The app-guided assessment of acetabular defects offers a promising innovative approach to simplify complex situations. It makes the challenging field of acetabular revision arthroplasty more approachable, especially for less experienced surgeons and offers insight and guidance in the planning stage as well as during the intra-operative process.

The digitalization of an acetabular defect classification through implementation of a web-based application is a valid approach to align the rating results and rating reproducibility of non-specialists to those of experienced orthopedic surgeons. The introduction of the ADC application will hopefully spawn further advances to make surgical planning more intuitive in the future.

## References

[1] Learmonth ID, Young C, Rorabeck C (2007). The operation of the century: total hip replacement. Lancet.

[2] Lübbeke A, Silman AJ, Barea C, Prieto-Alhambra D, Carr AJ (2018). Mapping existing hip and knee replacement registries in Europe. Health Policy.

[3] Paprosky WG, Perona PG, Lawrence JM (1994). Acetabular defect classification and surgical reconstruction in revision arthroplasty: a 6-year follow-up evaluation. J Arthroplasty.

[4] D’Antonio JA, Capello WN, Borden LS, Bargar WL, Bierbaum BF, Boettcher WG, Steinberg ME, Stulberg DS, Wedge JH (1989). Classification and mangement of acetabular abnormalities in total hip arthroplasty. Clin Orthop Relat Res.

[5] Gross AE, Allan DG, Catre M, Garbuz DS, Stockley I (1993). Bone grafts in hip replacement surgery. The pelvic side. Orthop Clin North Am.

[6] Engh CA, Glassman AH, Griffin WL, Mayer JG (1988). Results of cementless revision for failed cemented total hip arthroplasty. Clin Orthop Relate Res.

[7] Wirtz DC, Jaenisch M, Osterhaus TA, Gathen M, Wimmer M, Randau TM, Schildberg FA, Roessler PP (2020). Acetabular defects in revision hip arthroplasty: a therapy-oriented classification. Arch Orthop Trauma Surg.

[8] Carbonell X, Chamarro A, Oberst U, Rodrigo B, Prades M (2018). Problematic use of the internet and smartphones in university students: 2006–2017. Int J Environ Res Public Health.

[9] Hymas C (2018) A decade of smartphones: we now spend an entire day every week online. The Telegraph. https://www.telegraph.co.uk/news/2018/08/01/decade-smartphones-now-spend-entire-day-every-week-online/. Accessed 11 Apr 2020

[10] Haas M (2018) Smartphone-Markt: Konjunktur und trends. Bitkom. https://www.bitkom.org/sites/default/files/file/import/Bitkom-Pressekonferenz-Smartphone-Markt-22-02-2018-Praesentation-final.pdf. Accessed 11 Apr 2020

[11] Lopez-Fernandez O, Kuss DJ, Romo L, Morvan Y, Kern L, Graziani P, Rousseau A, Rumpf HJ, Bischof A, Gässler AK, Schimmenti A, Passanisi A, Männikkö N, Kääriänen M, Demetrovics Z, Király O, Chóliz M, Zacarés JJ, Serra E, Griffiths MD, Pontes HM, Lelonek-Kuleta B, Chwaszcz J, Zullino D, Rochat L, Achab S, Billieux J (2017). Self-reported dependence on mobile phones in young adults: a European cross-cultural empirical survey. J Behav Addict.

[12] Krebs P, Duncan DT (2015). Health app use among US mobile phone owners: a national survey. JMIR Mhealth Uhealth.

[13] Dittrich F, Back DA, Harren AK, Landgraeber S, Reinecke F, Serong S, Beck S (2020). Smartphone and app usage in orthopedics and trauma surgery: survey study of physicians regarding acceptance, risk, and future prospects in Germany. JMIR Form Res.

[14] Randau TM, Jaenisch M, Haffer H, Schömig F, Kasapovic A, Olejniczak K, Flechtenmacher J, Perka C, Wirtz DC, Pumberger M (2020). Collateral effect of COVID-19 on orthopedic and trauma surgery. PLoS ONE.

[15] Scheidt S, Kehrer M, Jaenisch M, Goost H, Wirtz DC, Burger C, Kabir K, Welle K, Wimmer MD (2020). A feasibility pilot study on the use of telemedicine for the examination of the knee joint. Z Orthop Unfall.

[16] Jaenisch M, Kohlhof H, Touet A, Kehrer M, Cucchi D, Burger C, Wirtz DC, Welle K, Kabir K (2020). Evaluation of the feasibility of a telemedical examination of the hip and pelvis—early lessons form the COVID-19 pandemic. Z Orthop Unfall.

[17] Hancock GE, Hepworth T, Wembridge K (2018). Accuracy and reliability of knee goniometry methods. J Exp Orthop.

[18] Landis JR, Koch GG (1977). The measurement of observer agreement for categorical data. Biometrics.

[19] Andrawis JP, Muzykewicz DA, Franko OI (2016). Mobile device trends in orthopedic surgery: rapid change and future implications. Orthopedics.

[20] Franko OI (2011). Smartphone apps for orthopaedic surgeons. Clin Orthop Relat Res.

[21] Kulendran M, Lim M, Laws G, Chow A, Nehme J, Darzi A, Purkayastha S (2014). Surgical smartphone applications across different platforms: their evolution, uses, and users. Surg Innov.

[22] Riouallon G, Sebaaly A, Upex P, Zaraa M, Jouffroy P (2018). A new, easy, fast, and reliable method to correctly classify acetabular fractures according to the Letournel system. JBJS Open Access.

[23] Wiechmann W, Kwan D, Bokarius A, Toohey SL (2016). There’s an app for that? Highlighting the difficulty in finding clinically relevant smartphone applications. West J Emerg Med.

[24] Campbell DG, Garbuz DS, Masri BA, Duncan CP (2001). Reliability of acetabular bone defect classification systems in revision total hip arthroplasty. J Arthroplasty.

[25] Nambiar M, West LR, Bingham R (2017). AO surgery reference: a comprehensive guide for management of fractures. BJSM.

[26] de Araujo H, Ono A, Chang VYP, Rodenbeck EM, Olveira de Araujo A, Gracia de Oliveira R, Marcon RM, FogacaCristante A, Filho TEPB (2020). Assessment of the accuracy of the AO Spine-TL classification for thorakolumbar spine fractures using the AO Surgery Reference Mobile App. Global Spine J.

